# Risk perception is unlikely to drive underuse of social information in lab studies

**DOI:** 10.3389/fpsyg.2026.1887755

**Published:** 2026-10-06

**Authors:** Patricio Cruz y Celis Peniche

**Affiliations:** Department of Anthropology, University of California, Davis, Davis, CA, United States

**Keywords:** epistemic vigilance, lottery choice experiment, risk, social information waste, social learning

## Abstract

Despite the theorized benefits of social learning, participants in controlled experiments consistently copy less than is optimal – instead over-relying on individual exploration. This may be due to learners’ perceptions of risk associated with social versus asocial information. To test this hypothesis, I recruited a large (N = 240) sample of UK adults to participate in an online experiment (‘The Farming Game’), in which they could maximize payoffs by accurately choosing which of two crops to grow over variable-yet-learnable environments. To measure participants’ risk-profiles, this study mirrored lottery-choice experiments widely used in behavioral economics. Participants were asked to choose between a ‘risky lottery’ and a ‘sure gain’ option on the basis of information they previously learned. How the information was learned varied across conditions (whether they were asked to copy socially or explore individually). However, the riskiness of the lottery choices themselves was held constant for all, such that any observed differences in risk-taking should speak to participants’ underlying priors regarding that source of information. Contrary to this study’s predictions, people assigned to social information were not significantly more (or less) risk averse than those assigned to asocial information. This suggests underuse of social information may not be due to how risky it is perceived.

## 1 Introduction

Early models of cultural evolution (Boyd and Richerson, 1985) suggest humans were naturally selected to be *opportunistic* and *selective* social learners – that is, to be choosy about *when* to copy and *whom* to copy. The ‘opportunistic social learners’ prediction states humans should learn socially when individual exploration is more costly or less accurate. Individual learning is still expected (these models predict an “evolutionary equilibrium amount of imitation”; Boyd and Richerson, 1995, p. 132) but only when the environment fluctuates and becomes too unpredictable. In a relatively stable environment, ‘scrounging’ on the efforts of others can be an adaptive way to discover optimal solutions (Barnard and Sibly, 1981). The ‘selective social learners’ prediction states humans should be biased imitators, preferentially copying some demonstrators over others rather than copying blindly or unbiasedly. These hypothesized social learning strategies [SLSs] include (1) content-based heuristics that directly bias learning based on how advantageous a cultural/behavioral trait seems (i.e., ‘copy-if-better *trait*’ strategies), and (2) demonstrator-based heuristics which indirectly bias learning based on specific aspects of a demonstrator (e.g., how similar, successful, or prestigious a demonstrator appears) (Henrich and McElreath, 2003; Laland, 2004).

Despite its theorized adaptive benefits (Boyd and Richerson, 1985; Henrich and McElreath, 2003), empirical studies put into question whether we make optimal use of our copying abilities. Participants in controlled lab experiments consistently underuse social information – instead over-relying on costly individual exploration (McElreath et al., 2005; Mesoudi, 2011; Morgan et al., 2012). This is true even when participants have access to additional knowledge on the quality of this information (e.g., knowing the behavior of the majority or the most successful individuals in these tasks). Morin et al. (2021) recently documented 45 instances of what they call ‘social information waste’ across five different experimental tasks. This kind of ‘egocentric discounting’ appears to contrast with widespread overimitation in the real world. That is, people appear to copy others beyond contexts that are useful. For example, prestigious individuals are commonly imitated for traits unrelated to their talent or success (e.g., athletes advertising underwear). This type of copying behavior has also been implicated in the global diffusion of fertility-reducing behaviors seen in the modern demographic transition – an extremely maladaptive phenomenon (Lu and Colleran, 2023). Why would humans under-use low-cost and easy-to-acquire social information inside the lab, while appearing to be enthusiastic overimitators in the real world?

The explanation to these apparently contradicting behaviors may lie with individuals’ perception of risk behind (a) social versus individual learning, and (b) between different kinds of social information. In a study replicating social information waste, Eriksson and Strimling (2009) suggested observed undercopying may be due to participants’ lack of trust in information produced by others, yet they did not formally test this hypothesis. This explanation is in line with Sperber et al. (2010) notion of ‘epistemic vigilance’, or Trouche et al. (2018) notion of ‘vigilant conservatism’. While too much vigilance (i.e., risk aversion) would seem to eliminate the benefits of social learning, not enough vigilance (i.e., risk proneness) can lead to maladaptive overimitation.

There are multiple reasons to mistrust social information, even independently of deceit. For one, SLSs themselves can be highly risky (Moya and Cruz y Celis Peniche, 2027). By definition, these cognitive heuristics are imperfect decision-making rules that evolved because they allow human learners to rapidly approximate local optima at a low cost, despite sometimes getting it wrong (Gigerenzer and Todd, 1999). Preferentially copying successful or prestigious models (i.e., indirect payoff bias; Henrich and Gil-White, 2001) may be a useful way to learn traits that could make one just as successful. Yet this strategy also carries the risk of learners copying a trait that is irrelevant (or even counter) to those models’ relative advantage (e.g., traditions become maladaptive; apparent competence or wealth could have been inherited rather than accrued). A content-based heuristic like direct payoff bias relies on more direct evidence of the relative benefits of copying a cultural trait. However, this still entails some risk: payoffs may be highly stochastic (Baldini, 2012), causally opaque (Henrich et al., 2015), or contingent on a high degree of similarity between the demonstrator and the learner (Smaldino and Velilla, 2025).

Despite the cognitive challenges social learners must navigate, is unclear whether social information is perceived as inherently riskier compared to individually sourced information, and if so, how learners weigh these risks subjectively, particularly when making decisions that are themselves risky or uncertain. On the one hand, cultural complexity is such that humans should, for some domains, consider relying on their own information-gathering abilities as riskier than relying on that of others (e.g., ethnobotanical or medical knowledge). However, how much we are willing to believe or act on this social knowledge may vary depending on the stakes. For example, we may be more likely to believe in, or act on, a friend’s recommendation for an action movie to watch or a restaurant to try (i.e., lower-variance and lower-cost decisions) than their recommendation for a travel destination or a tattoo artist.

This study aims to contribute to these open questions by testing the hypothesis that ‘social information waste’ observed in the laboratory is due to participants’ lack of trust in information produced by others. In particular, it explores whether a minimal form of social information is perceived as inherently riskier compared to individually sourced information in uncertain situations, under the assumption that people will respond to this type of risk by acting more risk-averse when exposed to the former.

To test this hypothesis, this study recruited a large sample of UK adults to participate in an online experiment (‘The Farming Game’), in which they could maximize payoffs by accurately choosing which of two crops to grow over variable-yet-learnable environments. While participants in this experiment were exposed to different sources of information (social vs. asocial), riskiness of choices was held constant across all learning conditions. Thus, any observed differences in participants’ willingness to take risks should speak to their underlying priors regarding the source of information available to them.

## 2 Methods

### 2.1 Participant recruitment and compensation

N = 80 adult participants were recruited for a pilot study and N = 240 adult participants were recruited for the main study. All participants were recruited through the virtual platform Prolific (prolific.com). Half of all participants were male and half were female. All of our participants were from the United Kingdom and had at least a high-school diploma as their level of schooling. Participants had to pass a visual search bot check to participate in the study. Participation in the pilot study was an exclusion criterion for participation in the main study. All participants were compensated at a base rate of $12 USD per hour, which equaled $4 USD for the pilot study, and $3 USD for the main study. Additionally, participants who scored above 80% correct in their responses were offered a bonus of $1 USD for the pilot study and $2 USD for the main study. All payments were handled through prolific. All data was collected in the months of February and March, 2026. All participants gave informed consent to participate in this study. This project was exempt from review by the Institutional Review Board of the University of California, Davis.

### 2.2 Farming game design

To measure participants’ risk-profiles, we designed an online experiment (‘The Farming Game’) using the virtual platform Gorilla (gorilla.sc), which can be easily integrated with Prolific. Our research design mirrored lottery-choice experiments widely used in behavioral economics (e.g., Baker et al., 2008), where participants are asked to choose between a ‘risky lottery’ and a ‘sure gain’ option. In particular, we exploit what Kahneman and Tversky termed the ‘Certainty effect’: the tendency for people to “overweight outcomes that are considered certain, relative to outcomes which are merely probable” (Kahneman and Tversky, 1979, p. 265). Similar to this experimental paradigm, participants in our study were asked to choose between a ‘risky lottery’ (henceforth ‘risky option’) and a ‘sure gain’ payoff (henceforth ‘certain option’). Unlike previous experiments of risk aversion, where participants are shown the probabilities of the risky lottery (e.g., 60% chance of winning $30 and 40% chance of winning $3), our task required participants to learn these probability distributions during a learning phase.

#### 2.2.1 Learning phase

Participants were given the option to sample two crops (labeled Barley or Wheat, counterbalanced), each with a binomial payoff distribution but different expected values (i.e., one was the ‘optimal’ crop, and the other was ‘suboptimal’; Supplementary Tables 1,2). To ensure that the expected value of each crop at the end of the learning phase was exactly the same across all conditions, we (a) controlled the exact payoffs each participant saw across seasons, randomizing only the order in which these values were shown; and (b) set a limit to how many times they could choose one crop over the other, such that they sampled each crop exactly ten times (i.e., 20 learning trials).

The way participants could learn the returns of these two crops varied depending on the learning condition they were randomly assigned to. Participants in both the pilot and the main study could be assigned to an (asocial) individual exploration [IE] or a (social) direct bias [DB] condition. The main study added another social learning condition, indirect bias [IB].

In the IE condition, participants could learn which of the two crops was better by trial-and-error (e.g., after choosing a crop, they were shown how many points that crop would give them during that trial). In the DB condition, participants were presented with a similar choice, only this time they were asked which of two players (i.e., demonstrators) they wanted to copy. Each player was described as preferring a different crop (i.e., either Barley or Wheat), and visually represented with an identical illustration so as to emphasize that any observed differences in payoffs was due to the trait and not a characteristic of the player (Figure 1). Participants were told these players were anonymous, and had been randomly assigned a name to make them easier to identify (i.e., Alex and Chris). After choosing a player, participants were then shown how many points that player earned in that trial. In the IB condition, participants were exposed to the same information as in the DB condition, yet each player was additionally shown next to an indirect cue of success. Five indirect cues were chosen to reflect different levels of wealth or success (i.e., in ascending order: a basket, a wheelbarrow, a cow, a truck, or an industrial harvester; Supplementary Material). The type of cue that appeared next to a demonstrator could vary across learning trials, and was determined by the running sum of that demonstrator’s crop yield, aggregated across all learning trials seen so far. This way to operationalize indirect bias allows us to recover some of its real-world properties (i.e., a prestigious demonstrator may continue to be preferentially imitated despite a sudden loss of payoffs).

**FIGURE 1:**
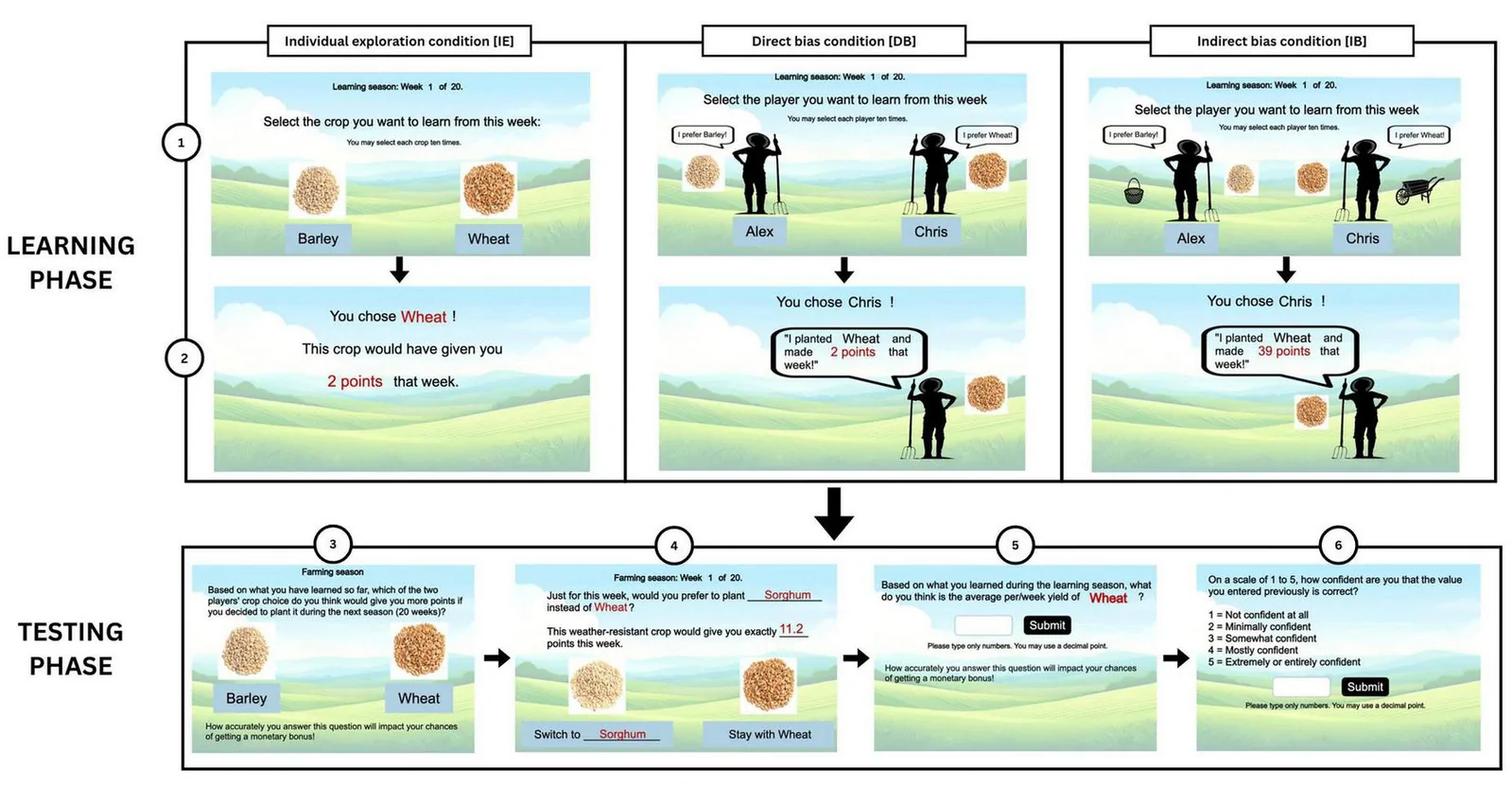
Screenshots of the Farming Game task. Steps 1 and 2 correspond to the learning phase of the experiment, and steps 3 to 6 to the testing phase. Participants were exposed to 20 trials of steps 1 and 2 on the learning phase, and 20 trials of step 4 on the testing phase. This example assumes a participant that chose Wheat as the best crop in Step 3.

#### 2.2.2 Testing phase

To begin the testing phase, participants were asked which of the two crops they were previously exposed to (i.e., during the learning phase) did they think was better overall or on average. Whatever they chose would constitute the ‘risky option’ for the rest of the testing phase (even if they chose the less optimal of the two learning-phase crops). The testing phase consisted of 20 trials in which participants had the opportunity to gain points by choosing between this crop (Option A; henceforth ‘risky option’) and a new kind of crop they hadn’t been previously exposed to (Option B; henceforth ‘certain option), which produced stable or sure gain returns they were told upfront (e.g., “this crop would give you $5 with 100% certainty if you choose to grow it this trial). In each trial, participants were offered a different ‘safe’ crop with a randomized label (e.g., sorghum, rice, corn, oats). Participants were told they would obtain similar returns as the farmer they chose to imitate (if they chose the ‘risky option’), and that their choice only applied for the current trial. Unlike the learning phase, participants could not see any information regarding the consequences of each of their choices after every trial. However, they were told those returns would count toward their final compensation (i.e., if they scored above 80% correct they would receive a monetary bonus).

At the end of the testing phase, participants were asked what they perceived the expected value of the crop they chose as the ‘risky option’ was. They were also asked how certain they were about this value on a 5-point Likert scale.

### 2.3 Adapted Holt-Laury task

Either before or after the Farming Game task (i.e., randomly counterbalanced), participants were also exposed to 20 trials of an adaptation of the Holt-Laury task (Holt and Laury, 2002). In it, participants were asked to choose between a risky lottery (‘risky option’; e.g., $10 with 20% chance and $2 with 80% chance) and a sure gain value (‘certain option’; e.g., $7 with 100% certainty) expressed in classic numerical form (Supplementary Table 3). Our task deviates from the canonical form of the Holt-Laury task in that the sure gain option is the same across trials and is essentially a fixed value rather than a lottery. Like in the original Holt-Laury task, participants were not given any feedback after each response, and the item order was randomized.

By having participants answer the lottery-choice task in numeric form, we aimed to have a well-established baseline measure of their individual risk-proneness, which we could then use to assess whether participants were understanding and accurately learning in the Farming Game task, and whether people are generally more risk averse when engaged with learning tasks than when they are given complete information about the lotteries directly.

### 2.4 Pilot study

Before our main study we conducted a pilot study with a smaller sample (N = 80), exposing participants to only two conditions (IE and DB). First, we wanted to ensure that participants could learn the lotteries adequately, given this is a novel implementation of the lottery choice experimental paradigm. Second, we wanted to know whether presenting participants with the adapted Holt-Laury task before the Farming Game would bias participant responses. Finally, we wanted to explore whether different magnitudes in payoffs (baseline and x5) influenced risk taking in our task. Results of this pilot study are presented in Section “4 Results”.

### 2.5 Controls

In addition to exposing all participants to the exact same payoffs for the two ‘risky option’ crops during the learning phase, our experiment aimed to make comparable the risk participants were exposed to during the testing phase. Lottery-choice experiments normally infer participants’ risk aversion by tracking their “crossover point from a low-risk to a high-risk lottery” (Baker et al., 2008, p. 367). In our learning experiment, while the ‘risky option’ is always equally risky (i.e., the probabilities of each crop’s payoff lottery are constant), we manipulated the reward for choosing the ‘safe’ or ‘sure gain’ crop (‘certain option’) across trials within the testing phase (Supplementary Tables 1,2). In doing so, we manipulated how risky one option is relative to another, changing the expected value of the ‘certain option’ relative to the expected value of the ‘risky option’. The values of the ‘certain option’ (for a participants’ given crop choice for the ‘risky option’) were (a) the same across conditions, (b) randomized across trials, and (c) intermediate relative to those of the ‘risky option’, such that they were never below or over the lowest or highest possible returns of the ‘risky option’, respectively.

Our study held constant the average complexity of all decisions involving a lottery across conditions and tasks. With this we hoped to minimize behavioral attenuation (Enke et al., 2024), and improve the reproducibility of our findings (Enke and Shubatt, 2024). Following Holt and Laury (2002), who found that participants are more risk-averse when the stakes are real and higher, we also controlled for the magnitude of payoffs participants are exposed to across all conditions (see Supplementary Tables 1,2 for a detailed description of all the values we used).

Our study also held constant the crop labels and illustrations across conditions in both the learning phase and the testing phase. During the learning phase, which of these two crops was optimal or suboptimal was counterbalanced within each condition. During the testing phase, the order and payoffs of the 20 “safe” crops was randomized. The labels and illustrations for demonstrators were also held constant across both social learning conditions. During the learning phase, which of these two demonstrator labels was associated with the optimal or suboptimal crop was counterbalanced within each condition. The illustrations of demonstrators were the same for both crops. Demonstrator labels and illustrations were intentionally chosen to be gender-neutral, to minimize noise in participant decision-making.

Finally, our experimental design also held the entertainment value (i.e., task engagement) constant across conditions. This is unlike previous learning experiments (e.g., Mesoudi, 2011) where participants only get to “play” during individual exploration – yet another hypothesis for social information waste.

### 2.6 Analytical approach

To analyze all data in this study we used the “brms” R package for Bayesian statistical modeling (Bürkner, 2017). To test a majority of our predictions we fit logistic regression models for binary outcomes. This type of model uses a logit link function to model the probability of a successful event, which in our case was whether a participant chose the ‘risky option’ or the ‘certain option’ across each of 20 test trials during the Farming Game task or the adapted Holt-Laury task, adjusting for the difference in the expected value of both options. For non-binary outcomes explored in the *post-hoc* analyses described in Section 2.7 we used a linear regression model (family = Gaussian). All regression models included random intercepts to account for repeated individual observations. Standard flat priors were used for all regression coefficients, and half student-t distribution priors were used for all intercepts and standard deviations in our models. All models used four chains, each with 2000 iterations, of which 1000 were used as warmup. Analyses were conducted using R Studio (Version 2026.01.1+403).

### 2.7 *Post-hoc* analyses

Three *post-hoc* analyses were conducted using data from our main study. The first of these consisted in evaluating how participant learning outcomes varied depending on the learning condition they were randomly assigned to during the learning phase of the experiment. Outcomes included in this analysis included the probability that participants accurately identified the optimal crop, the degree of error in their perception of the expected value of their chosen crop, and the optimality of their responses (based on the true expected value of this crop) (Supplementary Figure 2).

The second and third of these *post-hoc* analyses consisted of a replication of our main analysis using two different sub-samples. These re-analyses were intended as exploratory, to evaluate whether the degree of attentiveness of participants influenced our main outcome of interest.

Attentive sub-sample 1: In this *post-hoc* analysis, attentiveness was proxied by whether a participant accurately identified the optimal crop in the *learning phase* of the experiment (Supplementary Figures 3,4). N = 47 participants were excluded using this criteria, resulting in a subsample of N = 193 for this secondary analysis.

Attentive sub-sample 2: In this *post-hoc* analysis, attentiveness was proxied by the proportion of responses that were optimal in the *testing phase* of the analysis (Supplementary Figures 5,6). N = 94 participants were excluded using this criteria, resulting in a subsample of N = 146 for this third re-analysis.

## 3 Main predictions

Prediction 1: First, we predict the more certain participants are about the payoff distributions they learned in The Farming Game, the more risk prone they will behave across all conditions. This prediction tests an important assumption underlying the hypothesis that humans not only perceive social information as riskier, but that they respond to this uncertainty by acting more risk-aversely.

Prediction 2: Second, we predict that social information (DB or IB conditions) will be perceived as more uncertain than individual information (IE condition). Therefore, participants exposed to social information will behave more risk-averse than those exposed to individually-sourced information (i.e., asocial condition). This would be consistent with the observed under-use of social information in learning experiments, but challenge theories of human social learning psychology that highlight its adaptive value (i.e., Boyd et al., 2011).

Prediction 3: Third, exposure to indirect cues that are positively correlated with trait payoffs will increase the perceived certainty of social information. Therefore, participants will be less risk-averse in the Indirect Bias condition relative to those exposed to direct (i.e., trait) payoff cues only (DB condition). This would be consistent with the theorized value of indirect cues as allowing learners to approximate the expected payoffs of a trait when these are variable, making this source of social information potentially more attractive than direct cues (Boyd and Richerson, 1985, Chapter 8).

Prediction 4: Fourth, we expect risky behavior in the Farming Game to be correlated with participant’s risk proneness in the adapted Holt-Laury task.

Prediction 5: Finally, given aging is associated with lower risk-taking cross-culturally (Sercombe, 2014; Mata et al., 2016), we predict younger participants will take more risks across all conditions, as well as in the lottery-choice task, in line with prior experiments of risk-aversion across age groups (e.g., Mather et al., 2012).

Failing to find a difference between risk-taking or uncertainty across groups exposed to social or individual information may suggest differential entertainment value, rather than risk perception, may explain social information waste seen in previous laboratory experiments. In previously documented cases of social information underutilization, participants typically got to ‘play’ in the asocial learning conditions only, whereas entertainment conditions are held constant in this experiment. The implications of this potential null finding, along with all the predictions described above, were pre-registered prior to any data collection here: https://doi.org/10.17605/OSF.IO/ANCE7.

## 4 Results

### 4.1 Pilot study results

The order in which participants were presented with the Holt-Laury task (either before or after the Farming Game) did not seem to significantly affect risk-taking in the Farming Game, our main outcomes of interest (β = 0.01; SE = 0.04; Supplementary Figure 1). This order did not affect risk-taking in the adapted Holt-Laury task either (β = −0.09; SE: 0.05). Regardless, we decided to maintain this order counterbalanced for our main study. Similarly, risk-taking in either task did not significantly vary across the learning conditions participants were assigned to, in either the low-payoff or high-payoff magnitude groups, although there appeared to be directional support for our second prediction (Supplementary Figure 1). Given the low-payoff group maximized variance in responses, we decided to use low-payoffs only for the main study. Participants seemed to be able to understand the Farming Game task, answering optimally an average of 13.3 out of 20 times in the testing phase. However, practically half of the participants failed to accurately identify the optimal crop in the learning phase, which led us to revisit the way we phrased our instructions during the main study (Supplementary Table 4). Revised instructions included a statement highlighting the monetary bonus participants could receive if they correctly identified the better crop during the learning phase and encouraged participants to focus on which crop was more profitable on average, given that “the same crop may sometimes produce higher or lower yields” (Supplementary material).

### 4.2 Main study results

Contrary to our first prediction, participants that were more certain about the payoff distributions they learned during the learning phase of the main study did not take more risks during the testing phase (Figure 2A). In fact, the opposite trend seems more likely, although these effects were not significant. Even among more attentive participants (i.e., those that scored above average in the number of optimal decisions), more certainty seems to be associated with *less*, not more risk taking, despite also being statistically insignificant (Supplementary Figures 4,6). Social information was not significantly perceived as more (or less) uncertain than asocial information. While participants assigned to the DB condition were more certain than those assigned to the IE condition, participants assigned to the IB condition were the least certain (Supplementary Figure 3).

**FIGURE 2:**
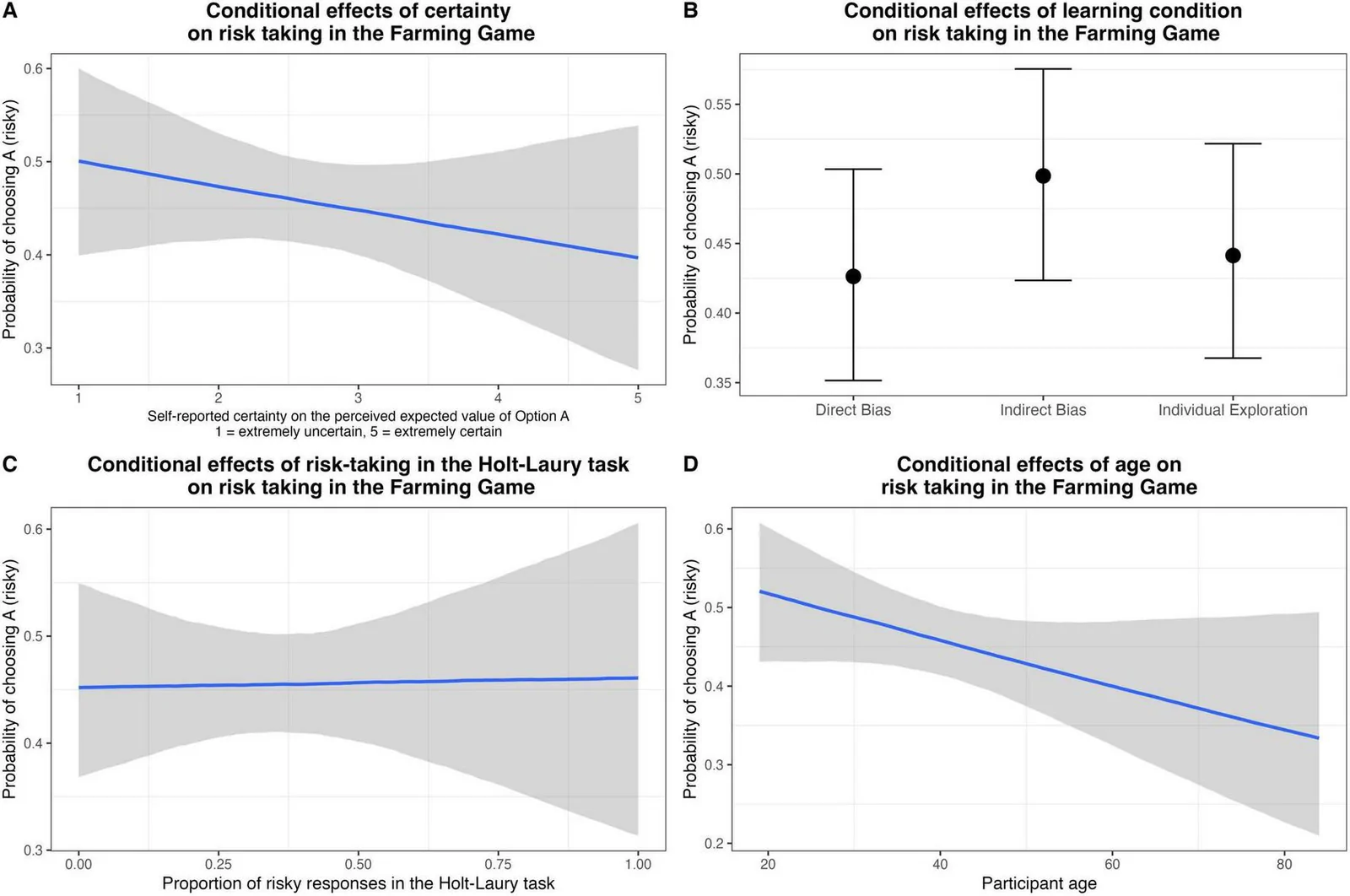
Conditional effects of **(A)** self-reported certainty, **(B)** learning condition, **(C)** risk-taking in the Holt-Laury task, and **(D)** age on risk-taking in the Farming Game. Bands and grey area represent 95% credible intervals.

Contrary to our second prediction, participants exposed to the social information conditions (DB and IB) did not consistently behave more risk averse (Figure 2B). While participants exposed to the DB condition took slightly less risks than participants exposed to the IE condition, participants exposed to the IB condition took the most risk out of all three groups.

Consistent with our third prediction, participants assigned to the IB condition made more risky decisions than participants assigned to the DB condition. However, these effects were not statistically significant, and participants exposed to the IB condition were actually *less* certain of the information they learned than those exposed to the DB condition (Figure 2B).

Contrary to our fourth and fifth predictions, participant risk-proneness was not correlated across the Holt-Laury task and the Farming Game task (Figure 2C), nor was it associated with younger age (Figure 2D). However, this may be an artifact of participant inattention. When we analyzed only the responses of more attentive participants (i.e., those that scored above average in the number of optimal decisions they made), we found that risk-taking in the Holt-Laury task and participant age both significantly predicted risk-taking in the Farming Game (Table 1; Supplementary Figures 6C,D).

**TABLE 1:** Estimated effects of our main predictor variables on our main outcome of interest in the Farming Game task (N = 240).

Predictions	Main analysis (full sample)	Attentive Sub-sample 1	Attentive Sub-sample 2
Prediction 1: Effect of self-reported certainty on risk-taking in the Farming Game	−0.11 [−0.33, 0.11]	−0.06 [−0.32, 0.19]	−0.10 [−0.39, 0.19]
Prediction 2: Effect of social learning conditions (DB/IB) on risk-taking different from effect of asocial learning condition (IE) on risk-taking in the Farming Game.	DB vs. IE: −0.05 [−0.50, 0.41] IB vs. IE: 0.24 [−0.18, 0.69]	DB vs. IE: 0.09 [−0.37, 0.56] IB vs. IE: 0.31 [-0.19, 0.81]	DB vs. IE: 0.19 [−0.42, 0.77] IB vs. IE: 0.41 [−0.18, 1.01]
Prediction 3: Effect of DB condition on risk-taking different from effect of IB condition on risk-taking in the Farming Game (i.e., DB vs. IB).	DB vs. IB: −0.29 [−0.73, 0.16]	DB vs. IB: −0.21 [−0.73, 0.30]	DB vs. IB: −0.24 [−0.82, 0.36]
Prediction 4: Effect of risk-taking in the Holt-Laury task on risk-taking Farming Game	0.01 [−0.94, 0.91]	0.09 [−0.91, 1.09]	**1.44 [0.23, 2.64]**
Prediction 5: Effect of age on risk-taking in the Farming Game	−0.01 [−0.03, 0.00]	−0.00 [−0.02, 0.01]	−**0.02 [**−**0.04,**−**0.01]**

Effects represent the median of the posterior distribution. 95% CI (Highest Density Intervals) are in brackets, and conventionally significant effects (also at the 95% level) are bolded.

While not a main goal of this study, random assignment to different types of social information presents a good opportunity to explore learning outcomes across these learning conditions. To recap, during the learning phase, participants were exposed to two crops with varying returns and later asked which of the two they thought was better, and what they thought the expected (i.e., average) value of whichever crop they chose was. This crop would become the ‘risky option’ in the testing phase.

Across all conditions, more participants chose the optimal crop than the suboptimal crop as ‘risky option’, and showed a significant improvement in this learning outcome relative to our pilot study (Supplementary Table 4). Those exposed to the DB condition correctly identified the optimal crop 85.4% of the time, followed by those exposed to the IE condition (79.6%) and those exposed to the IB condition (71.4%). This result did not significantly vary among more attentive participants (i.e., those that made above average optimal decisions during the testing phase; Supplementary Table 4).

When asked what they thought was the expected (average) value of the optimal crop they observed during the learning phase, participants exposed to social information (DB and IB conditions) gave estimated values almost twice as far from the true value compared to participants exposed to asocial information (IE condition) (Supplementary Figure 2B).

That being said, when considering their decisions based on the true expected value of the crop they chose as the ‘risky option’, there was essentially no difference in the extent to which participants behaved optimally across all three learning conditions in the testing phase (Supplementary Figure 2D).

The order in which participants completed the adapted Holt-Laury task (either before or after completing the Farming Game) did not significantly influence risk-taking in the Farming Game in our main study (β = −0.15; 95% CI: [−0.39, 0.09]), nor did this order influence risk-taking in the Holt-Laury task (β = −0.15; 95% CI: [−0.39, 0.09]). On average, participants took more risks in the Farming Game task than on the Holt Laury task, and these effects did not significantly change depending on the order in which participants completed both tasks (Supplementary Table 5).

All models used in the main analysis fit the observations reasonably well. All parameters in each model had an $\hat{\text{R}}$ (R-hat) of 1.01 or 1.00, and none of our four main models (i.e., those testing for the five main predictions) had any divergent transitions. Bulk effective sample sizes and tail effective sample sizes for each parameter of our four main models are reported in the Supplementary Table 6. Plotting standardized residuals versus the fitted values of our main models indicates some attenuation, such that participants were choosing A or B more often than our models would predict (left and right extremes of plot; Supplementary Figure 7). Including a covariate representing an item’s difference in the expected value of both possible choices greatly improved our models’ fit. Adding an item’s Objective Complexity Index (Enke and Shubatt, 2024) did not significantly improve models’ fit, so we excluded this term from our analysis. Posterior predictive checks on all of our main models indicate reasonable goodness of fit (Supplementary Figure 9).

## 5 Discussion

Participants did not behave in a way that supported the hypothesis tested by this study. When exposed to a particular and relatively minimal form of social information, participants do not appear to have perceived the riskiness of such information differently than that of asocial information.

There are multiple possible reasons behind this finding. The first one is that participants simply did not understand the Farming Game task. This concern seems reasonable given the relative novelty of our game implementation of the lottery choice paradigm. However, results suggest participants by and large understood the task at hand. First, the average proportion of optimal responses participants made in the Farming Game (14.8 out of 20) are comparable to – and even greater than – those made on the Holt-Laury task (13.4 out of 20). Second, the general distribution of these decisions follows the traditional S-shaped pattern seen in previous Holt-Laury task experiments, whereby participants tend to switch from choosing B (safe) to choosing A (risky), or vice versa, as the difference in the expected value of these two options decreases (Figure 3).

**FIGURE 3:**
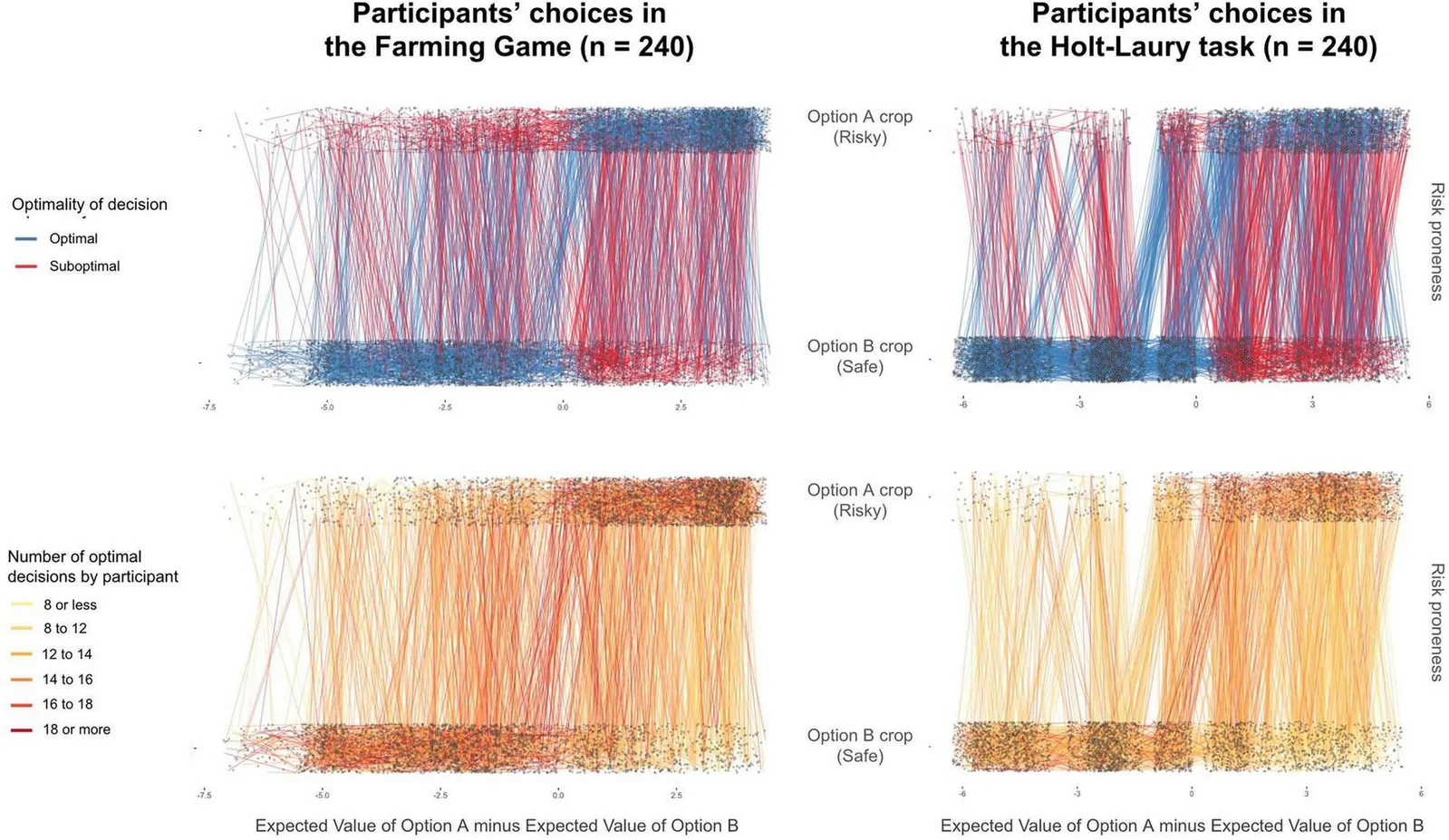
Aggregate decisions across 20 trials of *N* = 240 participants in the Farming Game (left column) and the Holt-Laury task (right column). Top row panels distinguish participant decision-making by the optimality of each decision (whether the chosen crop has a greater expected value than the alternative). Bottom row panels distinguish participant-decision making by the number of optimal decisions made by each participant (darker colored participants made more optimal decisions). Notice the shift in the tendency to choose one crop over the other at the point of payoff equivalence between both choices.

A second possibility is that participants do perceive social information as riskier, but respond to this information by being more risk *prone* than when exposed to asocial information. This would violate the assumption that participants will respond to greater perceived uncertainty by behaving more risk averse. The fact that participants assigned to the DB condition were both the most confident about the lotteries presented to them during the learning phase and the most risk-averse in the testing phase would suggest such a possibility. However, the fact that participants assigned to the other social condition (IB) took the most risks of all suggests otherwise.

Yet another possibility is that our experimental design failed to prime participants such that the social information conditions were not perceived as qualitatively different from the asocial information condition. In other words, despite telling participants in the social condition that they would “be able to evaluate how well crops do by looking at other players’ past performance”, participants did not perceive the information they were exposed to in this condition as social. This is possible given the minimal aspect of our social condition (e.g., demonstrators are anonymous, and there is no interaction with them whatsoever). However, this would seem inconsistent with the different directions of the effects of the Direct Bias condition (less risk than asocial condition) vis-à-vis the Indirect Bias condition (more risk than asocial condition). Despite these effects being statistically insignificant, they suggest that at the very least both social conditions may have been perceived differently from each other.

A final (and optimistic) explanation for these results seems to be that participants simply did not perceive social information, as a whole, as more risky than asocial information. If such biases exist, our experiment was not sensitive enough to capture those effects in a consistent nor significant way. On the other hand, if our experiment’s results are to be taken at face value, then the observed social information waste widely observed in laboratory studies may not be due to epistemic vigilance, but some other factor. In particular, the fact that our experiment held constant the entertainment value of our game across all three conditions would open the possibility that this is an important predictor of the way participants behaved in the social experiments reviewed by Morin et al. (2021).

Our findings also suggest that participants’ priors about the riskiness of social information is influenced by the presence of indirect cues. Contrary to the hypothesized value of indirect cues, exposure to these seemed to have worsened learning outcomes, be associated with decreased certainty, and increased sub-optimal risk taking in the IB condition group, relative to both the DB and the IE groups. In the absence of indirect cues, participants seemed to feel more confident about the information they learned from others (i.e., the DB condition) over the information they learned on their own (i.e., the IE condition); on the other hand, the addition of indirect cues seems to have slightly worsened participants’ confidence in how well they learned this information relative to the asocial condition. One limitation of these findings is that participants were not explicitly told the indirect cue was positively associated with the simultaneously observed demonstrator’s payoffs, to prevent participants from exclusively relying on this cue. Yet one of the assumptions of this hypothesized learning heuristic is that humans can evaluate both types of cues simultaneously – along with many others (e.g., similarity, frequency of traits, etc.). Making the payoffs associated with each decision or demonstrator simultaneously visible should have made this type of inference easier, yet the fact that learning outcomes worsened suggest maybe they added additional complexity or attention load.

These findings suggest that factors other than risk perception, such as differential entertainment value across learning conditions, may be more likely to explain social information waste seen in previous laboratory experiments. However, it is worth acknowledging that, while this study failed to find significant support for its predictions, it is possible that the way social information was operationalized was too minimal for us to detect an effect that may nonetheless be theoretically meaningful.

## Data Availability

The datasets presented in this study can be found in online repositories. The names of the repository/repositories and accession number(s) can be found below: https://osf.io/kebu9/files/osfstorage.
