# Supplementary material for: Risk perception is unlikely to drive underuse of social information in lab studies
Source: Front Psychol. 2026 Oct 6;17:1887755. doi: 10.3389/fpsyg.2026.1887755 (PMC13680137; doi:10.3389/fpsyg.2026.1887755)
Supplement: Supplementary file 1 [file Supplementary_file_1.docx]

Supplementary Materials for Cruz y Celis Peniche, P. (2026). Risk perception is unlikely to drive underuse of social information in lab studies

**Table S1. Complexity and values of payoffs used for the optimal crop in the testing phase of the Farming Game task in the main study (N = 240).** OCI = Objective complexity index (Enke & Shubatt, 2024). Average OCI = 0.27.

| **Trial** | **Option A** | **Option B** | **Optimal** | **OCI** |
| --- | --- | --- | --- | --- |
| 1  2  3  4  5  6  7  8  9  10  11  12  13  14  15  16  17  18  19  20 | A: 25 with 40% chance + 4 with 60% chance  A: 25 with 40% chance + 4 with 60% chance  A: 25 with 40% chance + 4 with 60% chance  A: 25 with 40% chance + 4 with 60% chance  A: 25 with 40% chance + 4 with 60% chance  A: 25 with 40% chance + 4 with 60% chance  A: 25 with 40% chance + 4 with 60% chance  A: 25 with 40% chance + 4 with 60% chance  A: 25 with 40% chance + 4 with 60% chance  A: 25 with 40% chance + 4 with 60% chance  A: 25 with 40% chance + 4 with 60% chance  A: 25 with 40% chance + 4 with 60% chance  A: 25 with 40% chance + 4 with 60% chance  A: 25 with 40% chance + 4 with 60% chance  A: 25 with 40% chance + 4 with 60% chance  A: 25 with 40% chance + 4 with 60% chance  A: 25 with 40% chance + 4 with 60% chance  A: 25 with 40% chance + 4 with 60% chance  A: 25 with 40% chance + 4 with 60% chance  A: 25 with 40% chance + 4 with 60% chance | B: 8.5 (with 100% certainty)  B: 8.8  (with 100% certainty)  B: 9 (with 100% certainty)  B: 9.2  (with 100% certainty)  B: 9.8  (with 100% certainty)  B: 10.3  (with 100% certainty)  B: 10.8  (with 100% certainty)  B: 11.2  (with 100% certainty)  B: 11.6  (with 100% certainty)  B: 12  (with 100% certainty)  B: 12.9  (with 100% certainty)  B: 13.4  (with 100% certainty)  B: 13.8  (with 100% certainty)  B: 14.2  (with 100% certainty)  B: 14.6  (with 100% certainty)  B: 15  (with 100% certainty)  B: 15.4  (with 100% certainty)  B: 16  (with 100% certainty)  B: 17  (with 100% certainty)  B: 17.4 (with 100% certainty) | A  A  A  A  A  A  A  A  A  A  B  B  B  B  B  B  B  B  B  B | 0.21 0.22 0.23 0.23 0.25 0.27 0.29 0.31 0.33 0.36 0.35 0.33 0.31 0.29 0.28 0.27 0.26 0.24 0.22 0.21 |

**Table S2. Complexity and values of payoffs used for the suboptimal crop in the testing phase of the Farming Game task in the main study (N = 240).** OCI = Objective complexity index (Enke & Shubatt, 2024). Average OCI = 0.28.

| **Trial** | **Option A** | **Option B** | **Optimal** | **OCI** |
| --- | --- | --- | --- | --- |
| 1  2  3  4  5  6  7  8  9  10  11  12  13  14  15  16  17  18  19  20 | A: 39 with 20% chance + 2 with 80% chance  A: 39 with 20% chance + 2 with 80% chance  A: 39 with 20% chance + 2 with 80% chance  A: 39 with 20% chance + 2 with 80% chance  A: 39 with 20% chance + 2 with 80% chance  A: 39 with 20% chance + 2 with 80% chance  A: 39 with 20% chance + 2 with 80% chance  A: 39 with 20% chance + 2 with 80% chance  A: 39 with 20% chance + 2 with 80% chance  A: 39 with 20% chance + 2 with 80% chance  A: 39 with 20% chance + 2 with 80% chance  A: 39 with 20% chance + 2 with 80% chance  A: 39 with 20% chance + 2 with 80% chance  A: 39 with 20% chance + 2 with 80% chance  A: 39 with 20% chance + 2 with 80% chance  A: 39 with 20% chance + 2 with 80% chance  A: 39 with 20% chance + 2 with 80% chance  A: 39 with 20% chance + 2 with 80% chance  A: 39 with 20% chance + 2 with 80% chance  A: 39 with 20% chance + 2 with 80% chance | B: 5.6  (with 100% certainty)  B: 5.7  (with 100% certainty)  B: 6.1  (with 100% certainty)  B: 6.3  (with 100% certainty)  B: 7  (with 100% certainty)  B: 7.4  (with 100% certainty)  B: 7.8  (with 100% certainty)  B: 8.2  (with 100% certainty)  B: 8.6  (with 100% certainty)  B: 8.8  (with 100% certainty)  B: 10.2  (with 100% certainty)  B: 10.6  (with 100% certainty)  B: 11.2  (with 100% certainty)  B: 11.8  (with 100% certainty)  B: 12.4  (with 100% certainty)  B: 13  (with 100% certainty)  B: 13.6  (with 100% certainty)  B: 14.2  (with 100% certainty)  B: 15  (with 100% certainty)  B: 16 (with 100% certainty) | A  A  A  A  A  A  A  A  A  A  B  B  B  B  B  B  B  B  B  B | 0.22 0.22 0.23 0.24 0.27 0.28 0.30 0.32 0.34 0.35 0.35 0.33 0.31 0.29 0.28 0.26 0.25 0.24 0.23 0.22 |

**Table S3. Complexity and values of payoffs used for the Holt-Laury task in the main study (N = 240).** OCI = Objective complexity index (Enke & Shubatt, 2024). Average OCI = 0.27.

| **Trial** | **Option A** | **Option B** | **Optimal** | **OCI** |
| --- | --- | --- | --- | --- |
| 1  2  3  4  5  6  7  8  9  10  11  12  13  14  15  16  17  18  19  20 | A: £2 with 80% chance + £39 with 20% chance  A: £5 with 60% chance + £31.25 with 40% chance  A: £2 with 80% chance + £39 with 20% chance  A: £10 with 60% chance + £62.5 with 40% chance  A: £2 with 80% chance + £39 with 20% chance  A: £5 with 60% chance + £31.25 with 40% chance  A: £2.5 with 80% chance + £48.75 with 20% chance  A: £5 with 60% chance + £31.25 with 40% chance  A: £2 with 80% chance + £39 with 20% chance  A: £5 with 60% chance + £31.25 with 40% chance  A: £2.5 with 80% chance + £48.75 with 20% chance  A: £4 with 60% chance + £25 with 40% chance  A: £2.5 with 80% chance + £48.75 with 20% chance  A: £4 with 60% chance + £25 with 40% chance  A: £2.5 with 80% chance + £48.75 with 20% chance  A: £4 with 60% chance + £25 with 40% chance  A: £2 with 80% chance + £39 with 20% chance  A: £4 with 60% chance + £25 with 40% chance  A: £2.5 with 80% chance + £48.75 with 20% chance  A: £4 with 60% chance + £25 with 40% chance | B: £5.6 (with 100% certainty)  B: £11.25 (with 100% certainty)  B: £7 (with 100% certainty)  B: £13.5 (with 100% certainty)  B: £8.6 (with 100% certainty)  B: £16 (with 100% certainty)  B: £11.2 (with 100% certainty)  B: £18.25 (with 100% certainty)  B: £13.6 (with 100% certainty)  B: £21.25 (with 100% certainty)  B: £6.75 (with 100% certainty)  B: £9 (with 100% certainty)  B: £8.75 (with 100% certainty)  B: £10.8 (with 100% certainty)  B: £10.75 (with 100% certainty)  B: £12.9 (with 100% certainty)  B: £14 (with 100% certainty)  B: £14.6 (with 100% certainty)  B: £17 (with 100% certainty)  B: £17 (with 100% certainty) | A  A  A  A  A  B  B  B  B  B  A  A  A  A  A  B  B  B  B  B | 0.22  0.23  0.27  0.10  0.34  0.37  0.31  0.29  0.25  0.22  0.21  0.23  0.27  0.29  0.35  0.35  0.32  0.28  0.26  0.22 |

**Table S4. Proportion of participants that accurately identified the optimal crop as Option A in the pilot study, the main study, and the attentive sub-sample (i.e., those that made above average optimal decisions in the testing phase of the main study).** 0 = chose the suboptimal crop; 1 = chose the optimal crop.

|  | **IE condition** | **DB condition** | **IB condition** |
| --- | --- | --- | --- |
| **Pilot study** | 0 = 52.6 % / 1 = 47.4 % | 0 = 42.9 %  / 1 = 57.1 % | NA |
| **Main study** | 0 = 20.4 % / 1 = 79.6% | 0 = 14.6 % / 1 = 85.4 % | 0 = 28.6 % / 1 = 71.4 % |
| **Attentive sub-sample** | 0 = 17.5 % / 1 = 82.5% | 0 = 12.7 % / 1 = 87.3 % | 0 = 28.4 % / 1 = 71.6 % |

**Table S5. Proportion of time participants chose Option A (risky) instead of Option B (safe) in both the Farming Game and the Holt-Laury task, by the order in which they completed these two tasks in both the pilot study and the main study.**

|  | **Pilot study** | **Main study** |
| --- | --- | --- |
| **Holt-Laury task first** | 0.52 | 0.46 |
| **Farming Game task first** | 0.51 | 0.49 |

**Table S6. Bulk effective sample sizes and tail effective sample sizes for each parameter in our four main models, which test for the main outcomes of interest (Predictions 1 through 5).**

| Fit 1: FG.Response.A ~ Condition + EV.of.A.minus.B.FG + (1 \| Participant.Public.ID) | | |
| --- | --- | --- |
| **Parameter** | **Bulk effective sample size** | **Tail effective sample size** |
| Intercept | 770 | 1265 |
| ConditionDB | 783 | 1270 |
| ConditionIB | 804 | 1470 |
| EV.of.A.minus.B.FG | 5950 | 3103 |
| Fit 2: FG.Response.A ~ How.certain + EV.of.A.minus.B.FG + (1 \| Participant.Public.ID) | | |
| **Parameter** | **Bulk effective sample size** | **Tail effective sample size** |
| Intercept | 1281 | 2067 |
| How.certain | 1261 | 1805 |
| EV.of.A.minus.B.FG | 8892 | 2858 |
| Fit 3: FG.Response.A ~ Age + EV.of.A.minus.B.FG + (1 \| Participant.Public.ID) | | |
| **Parameter** | **Bulk effective sample size** | **Tail effective sample size** |
| Intercept | 1007 | 1975 |
| Age | 1065 | 1876 |
| EV.of.A.minus.B.FG | 6159 | 2780 |
| Fit 4: FG.Response.A ~ Prop.A.HL + EV.of.A.minus.B.FG + (1 \| Participant.Public.ID) | | |
| **Parameter** | **Bulk effective sample size** | **Tail effective sample size** |
| Intercept | 907 | 1953 |
| Prop.A.HL | 1003 | 1825 |
| EV.of.A.minus.B.FG | 6934 | 2812 |

**Instructions to the Farming Game task shown to participants by condition (each condition had N = 80 participants)**

Individual Exploration [IE] - learning phase

“Your task as a farmer is to make the most points by choosing the best crop to grow throughout a farming season. All seasons are identical, and consist of 20 weeks. Correctly identifying the best crop will increase your chances of getting a monetary bonus at the end. Before farming, you will go through a learning season, where you will be able to evaluate how well crops do, without this impacting the points you make. Notice the same crop may produce higher/lower yields in different weeks due to weather changes throughout the season. To learn which crop is better, try to focus on which one yields more points on average.”

Direct Bias [DB] and Indirect Bias [IB] - learning phase

“Your task as a farmer is to make the most points by choosing the best crop to grow throughout a farming season. All seasons are identical, and consist of 20 weeks. Correctly identifying the best crop will increase your chances of getting a monetary bonus at the end. Before farming, you will go through a learning season, where you will be able to evaluate how well crops do by looking at other players’ past performance. This phase will not impact the points you make. The players are anonymous, but we have randomly assigned them names to make them easier to identify. Notice the same player may obtain higher/lower yields in different weeks due to weather changes throughout the season. To learn which player does better, try to focus on which one makes more points on average.”

All conditions - testing phase

“Now that you’ve had a chance to learn which of the two previous crops is best, you will go through a farming season also consisting of 20 weeks. This time, your decisions can impact your monetary reward, but you won’t know your score until the end of the game. Each week, you will be offered to plant [crop they chose as Option A]. You can expect to obtain similar yields as what you saw in the learning phase; that is, sometimes more, sometimes less. In addition to this familiar crop, you will now also be offered to grow a new kind of crop instead. This type of crop produces intermediate but stable yields. You will be told upfront exactly how much this alternative crop would give you that week. Whatever you decide to grow only applies to the current week, not the whole season.”

All conditions - Elicitation of perceived expected value and self-reported certainty

“Based on what you learned during the learning season, what do you think is the average per/week yield of [crop they chose as Option A]? Please type only numbers. You may use a decimal point. How accurately you answer this question will impact your chances of getting a monetary bonus.”

“On a scale of 1 to 5, how confident are you that the value you entered previously is correct? 1 = Not confident at all; 2 = Minimally confident; 3 = Somewhat confident; 4 = Mostly confident; 5 = Extremely or entirely confident. Please type only numbers”

**Instructions to the adapted Holt-Laury task shown to all participants (N = 240)**

“Below, you will be presented with a series of 20 choices, each consisting of two hypothetical scenarios (Options A and B). Your job is to indicate which of these two hypothetical options you would prefer if you were presented with them in the real world. **Option A** will always be a lottery. This means the reward you would get for this option  varies depending on chance. This chance will be expressed in terms of a percentage (e.g., a coin toss has 50% chance of landing *heads* + 50% chance of landing *tails*). **Option B** will always be a fixed value. Choosing this option would mean getting this reward with 100% certainty. This section is not timed, but taking less than 10s or more than 30s per question may impact your chances of getting a monetary bonus. While you will not be awarded the amounts in the options that you choose, how accurately you answer will impact your chances of getting a monetary bonus!”

**Figures used as indirect cues (i.e., success cues) in the Farming Game task of the main study (N = 240).** Figures positively covaried with the cumulative payoffs of each observed demonstrator.

| **Less than 25**  **points** | **Greater than 25 points** | **Greater than 50 points** | **Greater than 75 points** | **Greater than 100 points** |
| --- | --- | --- | --- | --- |
|  |  |  |  |  |
| **Basket** | **Wheelbarrow** | **Cattle plough** | **Truck** | **Harvester** |
| <- Less successful                                                                                                                      More successful -> | | | | |

**Figure S1. Conditional effects of learning condition and order on risk-taking in the pilot version of the Farming Game task (N = 80).** Bands represent 95% credible intervals.

**Figure S2. Conditional effects of learning condition on (A) accurate identification of the optimal crop as Option A, (B) deviations from the true expected value of Option A, (C) self-reported certainty, and (D) optimality of decision in the Farming Game.** Panels A to C represent outcomes from the learning phase of the experiment, whereas Panel D represents decisions in the testing phase of the experiment. Bands represent 95% credible intervals.

**Figure S3. Attentive Sub-sample 1, based on accurate identification of the optimal crop in the learning phase of the Farming Game task.** N = 47 participants in red (who misidentified the optimal crop) were excluded for re-analysis.

**Figure S4. Post-hoc analysis on Attentive Sub-sample 1 (N = 193), based on accurate identification of the optimal crop in the learning phase of the Farming Game.** Panels show conditional effects of (A) self-reported certainty, (B) learning condition, (C) risk-taking in the Holt-Laury task, and (D) age on risk-taking in the Farming Game. Bands and grey area represent 95% credible intervals.

**Figure S5. Attentive Sub-sample 2, based on optimal responses in the testing phase of the Farming Game task.** N = 94 participants in red (below the mean) were excluded for re-analysis.

**Figure S6. Post-hoc analysis on Attentive Sub-sample 2 (N = 146), based on optimal responses in the testing phase of the Farming Game.** Panels show conditional effects of (A) self-reported certainty, (B) learning condition, (C) risk-taking in the Holt-Laury task, and (D) age on risk-taking in the Farming Game. Bands and grey area represent 95% credible intervals.

**Figure S7. Scatterplots of Pearson (standardized) residuals, vs. fitted values for the four models used in our main analysis (N = 240).**

**Figure S8. Conditional effects of order of the tasks on risk-taking in the adapted Holt-Laury task (left) and the Farming Game (right) for our main dataset (N = 240).**

**Figure S9. Bar plots showing posterior predictive checks for all four main models using the main analysis to test for the five main predictions of this study (N = 240). These predict risk-taking based on (A) Learning condition; (B) Participant self-reported certainty; (C) Participant age; and (D) Risk-taking in the adapted Holt-Laury task. Bar plots compare the observed proportions/counts of 0s and 1s of our main outcome of interest (whether a participant chose A or not; *Y*) against each model's predicted counts (*Yrep*).**
